# Leaf-transcriptome profiles of *phoebe bournei* provide insights into temporal drought stress responses

**DOI:** 10.3389/fpls.2022.1010314

**Published:** 2022-10-24

**Authors:** Xiang Li, Lanlan Liu, Shixian Sun, Yanmei Li, Lu Jia, Shili Ye, Yanxuan Yu, Komivi Dossa, Yunpeng Luan

**Affiliations:** ^1^ The First Affiliated Hospital of Yunnan University of Traditional Chinese Medicine, Kunming, China; ^2^ Key Laboratory for Forest Resources Conservation and Utilization in the Southwest Mountains of China, Ministry of Education, Southwest Forestry University, Kunming, China; ^3^ Yunnan Key Laboratory of Plateau Wetland Conservation, Restoration and Ecological Services Southwest Forestry University, Kunming, China; ^4^ Department of Life Technology Teaching and Research, School of Life Science, Southwest Forestry University, Kunming, China; ^5^ Faculty of Mathematics and Physics, Southwest Forestry University, Kunming, China; ^6^ CIRAD, UMR AGAP Institut, Montpellier, France

**Keywords:** antioxidant enzyme activity, nanmu, leaf transcriptome, phytohormone signaling, photosynthesis, starch and sucrose, transcription factors, water withholding stress

## Abstract

*Phoebe bournei* (Hemsl.) Yang is used as a commercial wood in China and is enlisted as a near-threatened species. Prolonged droughts pose a serious threat to young seedlings (1-2 years old). A transcriptome sequencing approach, together with the measurement of growth parameters and biochemical analyses were used to understand *P. bournei’s* drought responses on 15d, 30d, and 45d of drought stress treatment. The stem and root dry weights decreased significantly with drought stress duration. Activities of antioxidative enzymes i.e., peroxidase (POD), superoxide dismutase (SOD), and catalase (CAT) increased significantly with the increase in drought stress duration. A total of 13,274, 15,648, and 9,949 genes were differentially expressed in CKvs15d, CKvs30d, and CKvs45d, respectively. The differential expression analyses showed that photosystem I and II underwent structural changes, chlorophyll biosynthesis, and photosynthesis were reduced. The genes annotated as POD, SOD, and CAT were upregulated in drought-treated leaves as compared to control. Additionally, plant-hormone signal transduction, MAPK signaling-plant, phenylpropanoid biosynthesis, flavonoid biosynthesis, and starch and sucrose metabolism pathways showed large-scale expression changes in major genes. We also found that members of 25 transcription factor families were differentially expressed. Our study presents and discusses these transcriptome signatures. Overall, our findings represent key data for breeding towards drought stress tolerance in *P. bournei*.

## 1 Introduction

*Phoebe bournei* (Hemsl.) Yang, commonly known as “nanmu”, is a commercial wood in China. It is mainly used as a decay-resistant wood in wood art, boat construction, and architectural work (Wang et al., 2021). It is naturally distributed in southern China (Ge et al., 2014). However, its abundant usage and afforestation have resulted in a reduction of populations that are now distributed to a range of habitats. Owing to this situation, *P. bournei* has been declared a near-threatened species, and therefore continued efforts are needed to protect this species (IUCN, 2013). With the increasing availability of information and data about this genus and particularly *P. bournei*, we are informed that regardless of its presence in areas with sufficient rainfall, the increasing and prolonged drought spells in summers are concerning the conservationists (Chen, 2010). Pilot studies and surveys have clearly indicated that water limitation can severely affect its growth (Ge et al., 2012; Ge et al., 2014).

Vascular plants respond to drought stress by involving complex regulatory mechanisms. These responses include a variety of cellular and molecular adjustments to adjust overall plant growth and survival. Changes in hydraulics, peptides, phytohormones, reactive oxygen species (ROS), and calcium signals are the early responses (Takahashi et al., 2020). Excessive light stress coupled with the changes in osmotic pressure in leaves initiate adjustments in light harvesting and subsequently in photosynthesis rate (Reddy et al., 2004). These stresses particularly mediate the changes in abscisic acid (ABA) and Jasmonic acid (JA) concentrations and resulting changes in the expression of ABA and JA responsive genes/pathways (Zhang and Huang, 2013). At the same time, the mitogen-activated protein kinase (MAPK) signaling cascade and ROS accumulation are activated, which affects guard cells and stomatal physiology (Atif et al., 2019). The generation of excess ROS causes oxidative damage and even cell death. Therefore, ROS should be controlled (through homeostasis) to a non-toxic level by ROS scavenging mechanisms. For this, plants have evolved efficient enzymatic and non-enzymatic antioxidative systems. Superoxide-dismutase (SOD), peroxidase (POD), catalase (CAT), and glutathione S-transferase (GST) are major ROS scavenging enzymes that are activated to detoxify ROS (You and Chan, 2015). In addition, the phenylpropanoid and flavonoid biosynthesis pathways are activated during abiotic stress conditions such as drought (Xu et al., 2021).

Earlier studies on *P. bournei* and/or related species have shown that drought stress threat significantly affects the seedlings in the early life span (first and the second year after planting) (Chen, 2010). Experiments on *P. bournei* seedlings have shown that malondialdehyde (MDA) content, CAT, SOD, and POD activities increased when stressed for 15-35 days. Similarly, water stress significantly affected the chlorophyll pigments (Ge et al., 2014). However, there is no information available on the key transcriptomic changes related to these biochemical and physiological changes. Though an attempt made to explore proteome changes in *P. bournei* leaves under drought stress (7-14 days) suggested that proteins were differentially expressed related to photosynthesis, metabolism, and defense (Lu et al., 2019). Nevertheless, the transcriptomic changes that occur in leaves upon withholding water for a prolonged period i.e., more than a month, are not yet explored. These changes are important for conservationists and breeders to identify/breed drought tolerant genotypes. Particularly, when we know that summers in southern China, where *P. bournei* populations are distributed, may prolong and drought can extend for more than a month (Bai et al., 2010). Considering this, the current study was planned to explore key differential transcriptomic changes in *P. bournei* leaves under prolonged drought stress conditions i.e., up to 45 days.

## 2 Material and methods

### 2.1 Plant material

Two years old *P. bournei* seedlings with uniform growth were selected as plant material. The seedlings were cultivated from seed on June 18^th^, 2019. The seeds were obtained from the Fujian Academy of Forestry, China. The experimental seedlings were grown in a greenhouse at the Restoration and Ecological Services Southwest Forestry University, Yunnan, China in pots with a diameter of 17 cm and height of 23 cm. The pots were filled with the field soil collected from a naturally growing *P. bournei* plantation site at Fujian, China. Throughout the two years, the seedlings were grown under standard agronomic practices. The average annual temperature of the experimental site was 15 °C. Our pilot experiments revealed that 50 days of drought causes 100% mortality. Therefore, in this experiment, we withheld water for 15, 30, and 45 days. Starting date of the experiment was July 1^st^, 2021. Before initiating the experiment, the seedlings were transferred to a waterproof plastic shed and were irrigated normally for a month. After that, the seedlings were divided into four groups i.e., control (CK), 15d, 30d, and 45d. The control group was watered normally to maximum field capacity, whereas, the other three treatment groups were not watered for the above-mentioned periods of time. Average temperature and humidity at the experimental site were 27°C and 78%, respectively. From each experimental group and CK, 10 plants were selected for further analyses.

### 2.2 Growth attributes measurements and biochemical analyses

Triplicate seedlings’ samples were harvested for each time point as well as CK, washed thoroughly and divided into three parts (roots, stems, and leaves). Leaves were washed thrice with distilled water and immediately stored in liquid nitrogen for RNA extraction and transcriptome analyses or were used for biochemical analyses. The roots and stems were fixed at 105 °C for 15 min followed by 12 hours of drying at 75 °C until a stable weight was achieved. Root dry weight (RDW) and shoot dry weight (SDW) were measured for the triplicate samples by using an electronic scale (RoHS, Huazhi Electronics, China).

Malondialdehyde (MDA) content of the leaves from the treatment groups and CK was measured according a modified method described earlier (Ge et al., 2014). Briefly, triplicate leaf samples from different plants of the same treatment group were collected and 0.5g of each was homogenized in 6% 2-thiobarbituric acid prepared in trichloroacetic acid. Homogenate was heated at 100 °C for 15 min, cooled, centrifuged (at 10,000g for 15 min), and supernatant was collected. The absorbance of the supernatant was then determined at 450, 532, and 600 nm in a Spectrophotometer (made, company, model, country). Finally, the MDA content was determined according to the following formulae and expressed as nmol/g of dry weight.

MDA = 6.45 x (A_532_ – A_600_) – 0.56 x A_450_

The activities of SOD, POD, and CAT in the leaves of CK and treatment groups were determined by using the commercially available test kits purchase from the Nanjing Jiancheng Bioengineering Institute (Nanjing, China) as reported earlier in [14 and references therein].

### 2.3 RNA extraction and illumina sequencing

Triplicate leaf samples stored in liquid nitrogen were used for RNA extraction and sequencing at Benagen Company Ltd., Wuhan, China (www.benagen.com). Total RNAs were extracted from 1g leaf samples by using TRIzol reagent. The purity of the extracted RNAs was checked on 1% agarose gels as well as by NanoPhotometer spectrophotometer (IMPLEN, Los Angeles, CA, USA). Quantification was done using a Qubit RNA Assay Kit in Qubit 2.0 Flurometer (Life Technologies, Carlsbad, CA, USA). The integrity of the extracted RNAs was assessed by using a RNA Nano 6000 Assay Kit for the Agilent Bioanalyzer 2100 system (Agilent Technologies, Santa Clara, CA, USA). After quality check, the RNAs were enriched with the oligodT and addition of the fragmentation buffer to interrupt them randomly. First strand cDNAs were synthesized, second strand synthesis was carried out, and double stranded cDNA was purified using AMPure XP beads. The ds-cDNA was end-repaired, A-tailed, ligated with sequencing adapters, and fragmented with AMPure XP beads. Size selection and PCR enrichment reactions were carried out to obtain final cDNA libraries followed by insert size and effective concentration tests. Libraries were then pooled and sequenced on Novaseq 6000 platform (Illumina, San Diego, CA, USA).

### 2.4 Sequenced data analyses

Sequencing error rate and GC content distribution were determined. The raw sequencing data was processed for quality. Reads with adaptors, paired reads, and low-quality reads were removed followed by BLAST (Johnson et al., 2008) to compared unigene sequences with annotation databases including Nr (Deng et al., 2006), Pfam (Finn et al., 2014), Swiss-Prot (Apweiler, 2001), KEGG (Kanehisa and Goto, 2000), and GO (Ashburner et al., 2000). The transcript sequences obtained from Trinity splicing were used as reference sequence and clean reads for each sample were analyzed by RSEM software (Li and Dewey, 2011). The featureCounts software (Liao et al., 2014) was used to calculate the FPKM value (expected number of Fragments Per Kilobase of transcript sequence per Millions base pairs sequenced) and expressed as boxplot. Principal Component Analysis (PCA) was computed in R. Differential gene expression between the desired sets of treatments was done in DESeq2 (Varet et al., 2016). After differential analysis, we used the Benjamini-Hochberg method to obtain False Discovery Rate (FDR). The differentially expressed genes (DEGs) were screened based on the criteria that if | log2 Fold Change| ≥ 1, and FDR < 0.05. The DEGs were then enriched on the KEGG pathways (Xie et al., 2011). Lastly, iTAK software was employed for the prediction transcription factors (TFs). The iTAK identifies TFs through HMM-HMM scan comparison using TF families from PlantTFDB and PlnTFDB (Zheng et al., 2016).

### 2.5 qRT-PCR analyses

The expression profiles of 13 P*. bournei* genes were studied in drought stress leaves on 15d, 30d, and 45d. The primers were designed in Primer3Plus (Untergasser et al., 2007) (**Table 1**). The transcripts were selected based on their interesting profiles in the photosynthesis, phytohormone signaling, starch and sucrose metabolism, and antioxidative activity pathways. We used a cDNA Reverse Transcription Kit (Applied Biosystem, USA) to reverse transcribe 100 ng of total RNA. A Qiagen PCR machine (Rotor-Gene 6000, Qiagen, Shanghai, China) was used for 20 μL reactions in triplicate for each gene (20 μL final volume). A house keeping gene (*tubulin*; GeneBank: AST35772.1) was used as a reference gene. The reaction mixture composition was as follows. Forward primer (5Mm) 0.8 µL, reverse primer (5Mm) 0.8 µL, SYBR ^®^ Premix Ex Taq (Tli RNaseH Plus) (2x) 10µL, DNA template 2.0 µL, and RNase Free dH_2_O 6.4 µL. The reactions were carried out under following conditions. Pre-denaturation at 30s at 95 °C, 20 °C/s (1 cycle), 5s at 95°C (40 cycles), 20 s at 60°C and 20°C/s, followed by the melting curve analysis (0s at 95°C, 20°C/s, 15s at 65°C, 20°C/s, 0s at 95°C, and 0.1°C/s. The correlation between the qRT-PCR data and respective FPKM values was computed using cor function in R.

**Table 1:** List of primers used for qRT-PCR analyses.

Gene ID	Forward primer sequence	Reverse primer sequence
*TR2173_c0_g1:* *Ferredoxin*	GGCAGGAGAACCAATC	CAAGCCATCACACTCAT
*TR46817_c0_g1:* *photosystem II oxygen-evolving enhancer protein 1*	TCAGCCACCGCATTCA	AATTAATTGCCCAAGCC
*TR10690_c0_g2:* *BRI1 kinase inhibitor 1*	TCTGCACTGTAAACGCTG	AATCGTTGTCCCTCCC
*TR37083_c0_g1:* *abscisic acid receptor PYR/PYL family*	AGAGGAGTGTCACTAA	CACTGAGGTCAGCACC
*TR5417_c0_g1:* *protein phosphatase 2C*	AGACTCCGGCTAGGAT	AGCAACTACGCAAGGA
*TR23437_c0_g1:* *transcription factor TGA*	CCAGACACCCATAGA	CTCAAGAAAGCAGGGT
*TR271_c0_g2:* *alpha-amylase*	TCCGCTAGTATTCCTGCTT	CGATGAATGCGGTGGC
*TR7478_c0_g1:* *ADP-sugar diphosphatase*	ATGGGAGGAGGACACG	TCCACTCTATGCCACCTCT
*TR65289_c0_g1:* *beta-glucosidase*	ATGAGGACCCTGCTTT	TCATTAACATCATTGCGA
*TR46352_c1_g1:* *granule-bound starch synthase*	TGCCAGTCAGACCAACG	CCGATCCTAGTATGGCAA
*TR17000_c0_g1:* *peroxidase*	TCGGGCTAGACCACAGT	AGCCAACCACCATCA
*TR5719_c2_g1:* *superoxide dismutase*	CCCACTGAGGAGCACAC	AAGTTGGGATTGGTTCT
*TR35209_c1_g1:* *catalase*	CTGGGAGGGACAACGA	CTTGATTGAACCACCCTA
*Tubulin*	ATGCCAACAGTGTTGA	ATGACGGCACAGAGGC

### 2.6 Statistical analysis

Data for SDW, RDW, MDA content, and the activities of CAT, POD, and SOD were presented as mean values of three replicates. The differences between the treatments for each tested parameter were tested by one-way analysis of variance using Microsoft Excel 2019 ^®^. The same software was used to calculate standard deviation and to perform least significance difference test at the 0.05 significance probability level.

## 3 Results

### 3.1 Shoot and root dry weights decrease with the prolongation of water deficit period

**Figure 1:**
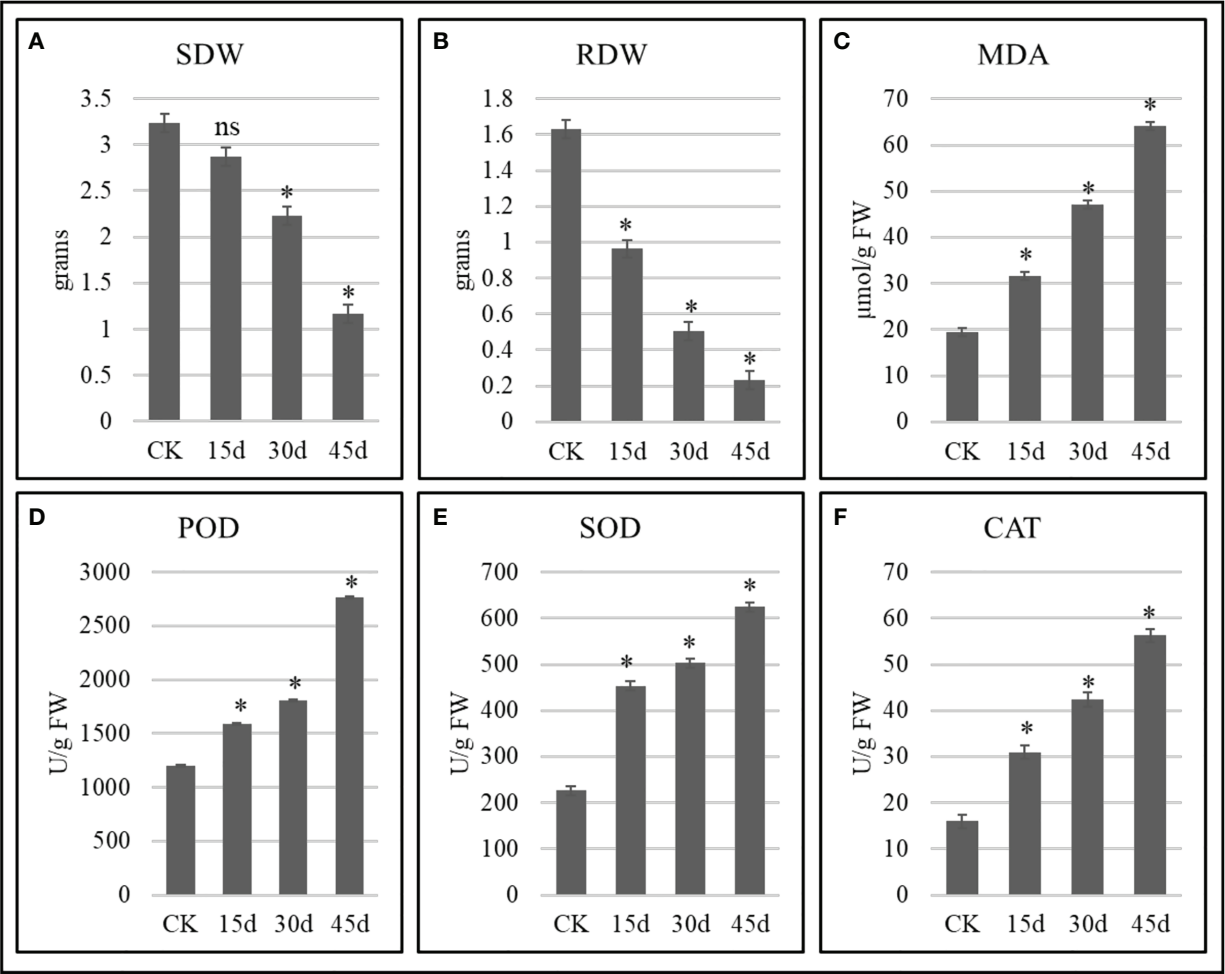
Growth and biochemical attributes of drought stressed *P. bournei* seedlings. **(A)** Stem dry weight, **(B)** root dry weight, **(C)** malonaldehyde, **(D)** peroxidase, **(E)** superoxide dismutase, and **(F)** catalase. The bars represent means values of three replicates ± standard deviation. ns and * indicate that treatment is non-significantly different and significantly different as compared to CK when *p < 0.05*, respectively.

**Figure 2:**
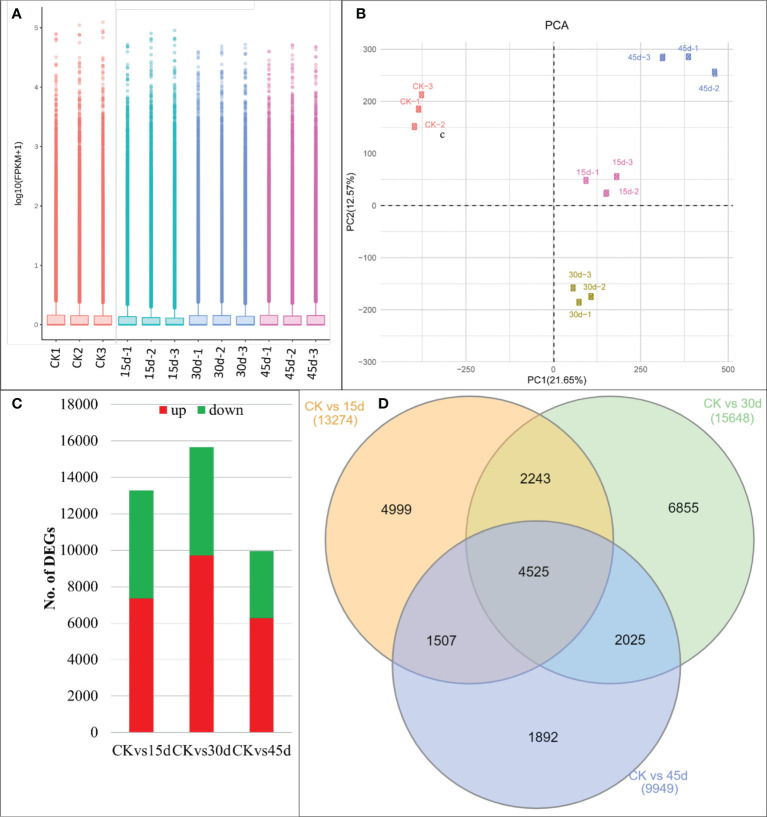
Transcriptome analyses of *P. bournei* leaves challenged with drought stress. **(A)** Distribution of the gene expression and **(B)** Principal Component Analysis. **(C)** Number of DEGs (up and downregulated) between the three treatment comparisons. **(D)** Venn diagram showing common and specific number of DEGs in the three treatment comparisons. Where, CK, 15d, 30d, and 45d represent control, and drought for 15, 30, and 45 days respectively. Numbers with the treatments represent replicates.

The growth attributes changed significantly in drought stressed *P. bournei* seedlings as compared to CK. The SDW decreased with the increase in days of stress. The non-significant differences between CK vs 15d for SDW indicate that the stress impacts are not evident in stem at this time. On the other hand, the significant differences between CK and 30d and 45d indicate that the stress significantly affects the stems if prolonged to 30d and 45d (**Figure 1**). Similarly, the significant reduction in RDW with time indicate that if stress is prolonged the negative impacts are intensified (**Figure 2**).

### 3.2 Changes in MDA content and antioxidant enzymes

The MDA increased significantly from 31.51 to 64.07 µmol/g FW after 15-45 days as compared to CK (19.33 µmol/g FW) (**Figure 1C**). On the contrary, we observed a rapid increase in the antioxidant enzyme activities i.e., POD, SOD, and CAT (**Figures 1D, E**). The antioxidant enzyme activities increased significantly with the prolongation of the drought stress and reached maximum on 45d. These observations indicate that with the increase in drought days, the *P. bournei* seedlings undergo increasing oxidative damage (as evident from MDA content changes) and the protective antioxidant mechanisms are activated which are manifested as an increased enzyme activity (**Figures 1D, E**).

### 3.3 Transcriptome profile of water stressed *P. bournei* leaves

The 12 libraries produced an average of 66.23 million clean reads (min. 50.9 and max. 80.5 million reads). 66.98% of the clean reads could be mapped onto the reference sequence (the transcripts after Trinity assembly and de-redundancy were used as the reference sequence). The error rate and GC contents were 0.03 and 44.193%, respectively (**Supplementary Table 1**). The sequencing produced 365,977 unigenes; which could be annotated to Nr, SwissProt, Pfam, GO, and KO (**Supplementary Figure 1**). The FPKM values for treatment replicates are presented in **Figure 2A**. The PCA analysis showed that the replicates for the same treatment were grouped thus the sampling was reliable. PC1 and PC2 explained 21.65% and 12.57% variation, respectively (**Figure 2B**).

### 3.4 Differential gene expression between the treated and control *P bournei* leaves

There were 13,274, 15,648, and 9,949 DEGs in CKvs15d, CKvs30d, and CKvs45d, respectively (**Figure 2C**). Of these, 4,425 DEGs were common to all treatment comparisons (**Figure 2D**). The DEGs in CKvs15d were significantly enriched in phenylpropanoid biosynthesis (ko00940), plant-pathogen interaction (ko04626), flavonoid biosynthesis (ko00941), starch and sucrose metabolism (ko00500), and plant hormone signal transduction (ko04075) pathways (**Supplementary Figure 2**).

### 3.5 Key differential transcriptomic signatures in drought stressed *P. bournei* leaves

#### 3.5.1 Chlorophyll and Photosynthesis related transcriptomic changes

Two light-harvesting complex I chlorophyll a/b binding proteins (LHCA1 and LHCA2) and light-harvesting complex II chlorophyll a/b binding proteins (LHCB1, LHCB2, and LHCB4) were differentially expressed in the studied treatments (**Supplementary Table 2**). The LHCA1 (*TR77177_c1_g1*), and LHCB4 (*TR5880_c1_g1*) were downregulated in all treatments as compared to CK. Interestingly, all antenna proteins (except LHCB1 and 4) were upregulated on 30d. Only LHCB1 and LHCB4 were upregulated on 45d. These changes indicate that if drought stress prolongs, the photosystem I and II (PSI and PSII) experience significant changes.

The porphyrin and chlorophyll biosynthesis genes were mostly upregulated on 30d. Only magnesium-protoporphyrin O-methyltransferase (bchM, *TR9676_c0_g1*), chlorophyllide a oxygenase (CAO, *TR4898_c0_g2*), 7-hydroxymethyl chlorophyll a reductase (HCAR, *TR14894_c0_g1*), and frataxin (FXN, *TR1277_c0_g1*) were upregulated on 15d. Similarly, on 45d a limited number of genes in this pathway were upregulated; including a heme oxygenase 1 (HMOX1, *TR10611_c0_g1*), three glutamate-1-semialdehyde 2,1-aminomutase (hemL) transcripts, and a HCAR (*TR14894_c0_g1*). These changes indicate that prolongation of drought stress to 30d activates a larger number of genes associated with porphyrin and chlorophyll metabolism and on 45d these changes are reduced.

The above observations were further strengthened by the observed changes in a large number of genes enriched in photosynthesis pathway; PSI, PSII, electron transport chain (ETC), and F-type ATPase related genes were differentially expressed. Among the ETC related DEGs, a ferredoxin (petF) transcript was downregulated in all treatments. F-type H+/Na+-transporting ATPase subunit alpha (atpA), F-type H+-transporting ATPase subunit b (atpF), a photosystem I subunit V (psaG), photosystem II 22kDa protein (psbS), photosystem II oxygen-evolving enhancer protein 1 (psbO), photosystem II oxygen-evolving enhancer protein 2 (psbP), and photosystem II oxygen-evolving enhancer protein 3 (psbQ) transcripts were downregulated in drought treated seedling leaves as compared to CK. These changes suggest a reduction in photosynthesis is a drought stress response in *P. bournei*.

#### 3.5.2 Effect of drought on phytohormone and MAPK signaling

A large number of DEGs (132) were enriched in plant-hormone signal transduction pathway indicating large-scale changes in hormone perception and signaling. The AUX1 (auxin influx carrier) transcripts were downregulated on 45d but showed mixed expression on other days. Most IAA transcripts were downregulated on 15d, 30d, and 45d. However, we noticed that the number of IAA and ARF transcripts that were downregulated on 30d and 45d reduced as compared to 15d, whereas the upregulated IAA transcripts increased on 30d and 45d. Two GH3 and SAUR proteins were downregulated while all others were upregulated in drought treated seedlings as compared to CK. The downregulation of histidine-containing phosphotransfer protein (AHP), ARR-B, and ARR-As indicate that drought stress lead towards the reduced cytokinin perception (Hutchison et al., 2006) and therefore seedling growth was restricted. Regarding gibberellins (GAs), three, one, and one DELLAs were downregulated in 15d, 30d, and 45d as compared to CK. Two phytochrome-interacting factor 3 (PIF3) and a PIF4 transcripts showed decreased and increased expressions in all drought treatments, respectively. The ABA responsive element binding factor (ABF), protein phosphatase 2C (PP2C), and serine/threonine-protein kinase SRK2 (SnRK2) were downregulated while the abscisic acid (ABA) receptor PYL were upregulated in drought treated seedlings as compared to CK. The upregulation of the genes/transcripts enriched in ethylene signaling further confirm the possibility of senescence. Whereas the brassinosteroid (BR) signaling related DEGs were downregulated. A JAR1 and JAZ were upregulated in 15d. Whereas, on 30d, a two JAZ’s showed opposite expressions as compared to CK. Similarly, JAR1 transcripts showed opposite expression patterns as compared to CK and the only MYC2 was exclusively upregulated in 30d. On 45d, JAR1 was downregulated and JAZ was upregulated. These changes indicate different JA signaling responses in studied treatments. As for salicylic acid (SA), two NPR1 were downregulated on 15d and 45d, while on was upregulated in 30d and 45d each. Except one PR1 (which was downregulated in all treatments), others were upregulated. Finally, TGAs were upregulated in drought treatments as compared to CK except one transcript that was highly downregulated in 15d (**Supplementary Table 2**). Thus, drought induces SA signaling in *P. bournei*.

In addition to phytohormone signaling related genes, major observations were the activation (upregulation) of calmodulin (CALM), mitogen-activated protein kinase 3 (MAPK3), and respiratory burst oxidase (RBOH) in drought treated seedlings. These changes indicate that *P. bournei* initiate a cascade of signaling when affected by drought.

#### 3.5.3 Starch and sucrose metabolism related transcriptomic changes in response to drought stress

**Figure 3:**
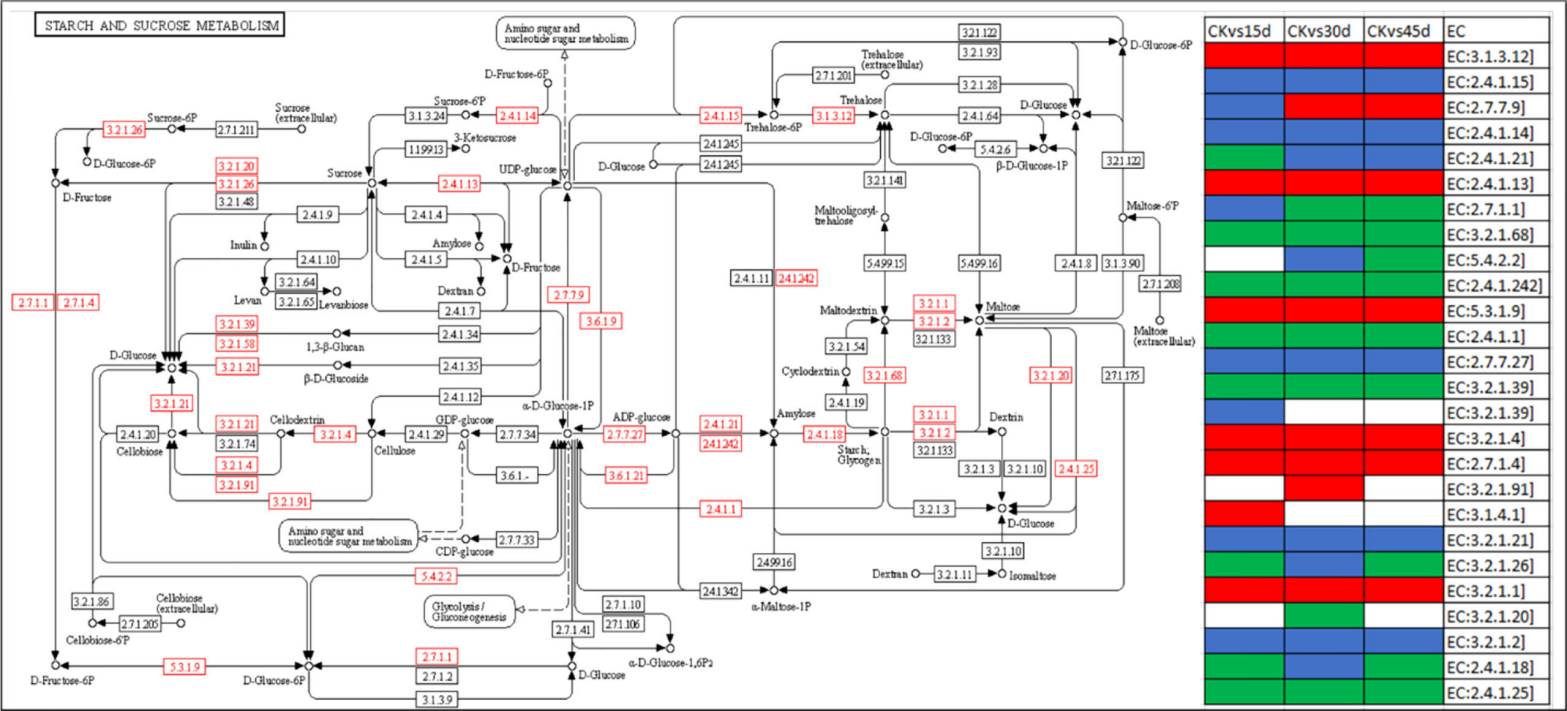
Differential regulation of starch and sucrose metabolism pathway. The red boxes show the DEGs. The heatmap panel on the right show expression trends of the enzymes, where green, red, blue, and white colors represent downregulated, upregulated, up/downregulated, and no expression, respectively. The pathway map was developed in the KEGG pathway database (Kanehisa, [[NoYear]]) using the KEGG pathway mapper (Kanehisa and Sato, 2020) by searching Ko IDs of the DEGs. The EC annotation details are given in the **Supplementary Table 2**.

Among the metabolism related pathways, 165 DEGs were enriched in starch and sucrose metabolism pathway. A large number of beta-glucosidase transcripts were upregulated in drought treated seedlings; some transcripts were specific while some were common. Additionally, alpha amylase (AMY), ADP-sugar diphosphatase (NUDX14), endoglucanase (EGL), fructokinase (scrK), starch synthase (glgA), sucrose synthase (SUS), trehalose 6-phosphate phosphatase (otsB), and UTP-glucose-1-phosphate uridylyltransferase (UGP2) were upregulated in drought treated seedlings as compared to CK. Whereas, 1,4-alpha-glucan branching enzyme (GBE1), beta-fructofuranosidase (INV), glucan endo-1,3-beta-glucosidase 1/2/3 (GN1_2_3), glycogen phosphorylase (PYG), and granule-bound starch synthase (WAXY) were downregulated in drought treated seedlings (**Figure 3**). These changes indicate that metabolic resources such as starch and sucrose are altered in stressed conditions for plant survival.

#### 3.5.4 Transcriptomic changes in secondary metabolites’ biosynthesis (phenylpropanoids and flavonoids biosynthesis)

The DEGs were significantly enriched in two secondary metabolite biosynthesis pathways i.e., phenylpropanoid biosynthesis (142) and flavonoid biosynthesis (43). The phenylpropanoid biosynthesis related transcripts were annotated as 15 genes. Of 98 transcripts, 23 were CKvs15d specific and showed increased expression as compared to CK. These included cinnamyl-alcohol dehydrogenase (CAD), peroxidase (POD), beta-glucosidase (bglX), 5-O-(4-coumaroyl)-D-quinate 3’-monooxygenase (C3’H), and shikimate O-hydroxycinnamoyltransferase (HCT). Interestingly, five detected phenylalanine ammonia-lyase (PAL) transcripts were downregulated in three treatments as compared to CK, while one PAL (*TR2133_c0_g1*) was upregulated in 30d and 45d. Although downregulated, the relative PAL expression in the drought treatments was increased from 15d to 30d and again decreased on 45d. Mixed expression patterns were noted for most of other genes i.e., a higher number of transcripts were upregulated in drought treated seedlings while a fraction of transcripts was downregulated e.g., CAD, COMT, and bglX. Whereas, coniferyl-aldehyde dehydrogenase (REF1) and HCTs were upregulated in drought treated seedlings. The DEGs enriched in flavonoid biosynthesis pathway showed variable gene expression. The expressions of the genes related to anthocyanin increased with the prolongation of drought stress but still remained lower than CK. Some chalcone synthases (CHS), flavonol synthases (FLS), C3’H, and HCTs were upregulated. However, most of these upregulated transcripts did express in 30d. Overall, these expression trends indicate that phenylpropanoid and flavonoid biosynthesis pathways experience large-scale expression changes in drought stress.

#### 3.5.5 Antioxidant system related genes (CAT, POD, SOD)

**Figure 4:**
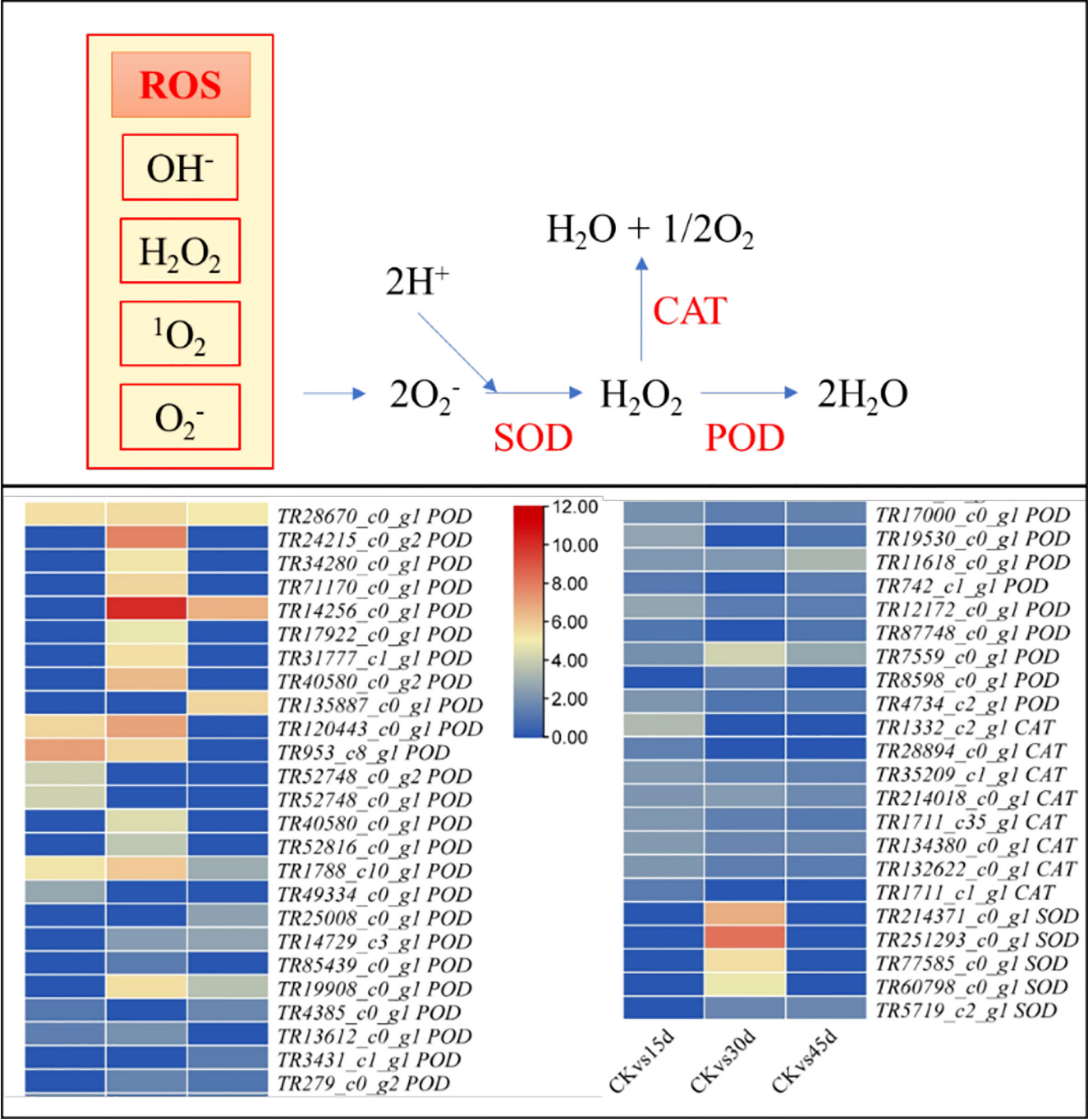
Differential changes in antioxidant enzymes (superoxide dismutase, SOD; catalase, CAT; peroxidase, POD) in *P. bournei* leaves challenged with drought stress for 15d, 30d, and 45d. Heatmap panels show log2FC values of the upregulated genes in drought treated leaves as compared to control (CK).

Four POD and three SOD2 didn’t express in CK as compared to drought treatments indicating that these are drought stress-induced genes. The differential expression showed that 18 and 8 PODs and CATs were upregulated in 15d, respectively, as compared to CK. Five PODs and three CATs were exclusive to 15d as compared to 30d and 45d. Similarly, 26, 5, and 5 POD, CAT, and SOD (SOD1 and SOD2) transcripts showed increased expressions in 30d as compared to CK. Ten POD and four SOD2 transcripts were exclusive to 30d as compared to 15d and 45d. On the other hand, 18, five, and one POD, CAT, and SOD1 transcripts were upregulated in 45d as compared to CK. Only three POD transcripts were specific to 45d as compared to 15d and 30d (**Figure 4**; **Supplementary Table 2**). These expression changes further confirm the POD, CAT, and SOD activity results that under drought stress the antioxidant enzymes are activated or their expression are increased.

#### 3.5.6 Transcription factors

The results showed that 194, 204, and 159 TFs were differentially expressed between CKvs15d, CKvs30d, CKvs45d, respectively. These TFs were classified into 25 families. Interestingly, WRKY6, 7, 24, 28, 40, 48, 53, 57, 65, 75, and 76 transcripts were upregulated in three drought treatments as compared to CK. While other WRKYs showed variable expression patterns. A large number of TFs classified as AP2, HLH, no apical meristem (NAM), and homeobox domain containing family members were differentially expressed (**Supplementary Table 3**). Seventeen AP2 domain containing TFs annotated as ethylene-responsive TFs were not expressed in 15d but upregulated on 30d and 45d (eight transcripts) as compared to CK. Whereas, five additional AP2 domain containing TFs were exclusively upregulated on 45d. All the B3 DNA binding domain containing TFs were downregulated in all treatments. The bZIP, CCAAT-binding, GATA zinc finger, HLH, SBP domain, TCP, Dof, GRF zinc finger, homeobox domain, and NAM TFs showed variable expression patterns within and across the treatments. Interestingly, the NAM83, 87, 90, 25, and 56 TFs were upregulated in drought treatments as compared to CK. Whereas some NAM TFs (104, 14, 22, 71, and 90) were 30d specific, while NAM37, 30, 68, and 43 were 45d specific. These changes indicate large scale transcriptional regulators are activated/deactivated in response to drought stress.

### 3.6 qRT-PCR analysis

**Figure 5:**
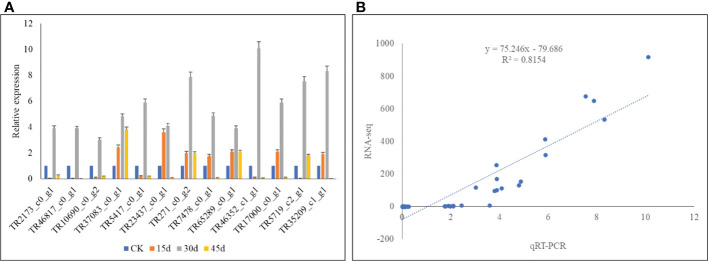
qRT-PCR analysis of the *P. bournei* genes in drought stressed leaves on 15d, 30d, and 45d. **(A)** Relative gene expressions and **(B)** correlation between relative gene expression and FPKM values of the selected genes.

The qRT-PCR analysis showed that the expression profiles of the selected genes were similar to that of RNA-seq results. This was confirmed by the observation that the R2 between the two datasets was 0.82. These results indicate the reliability of the RNA-seq results (**Figure 5**).

## 4 Discussion

### 4.1 Reduction in stem and root dry weight is a common drought stress response in *P. bournei*

In this work, the transcriptome sequencing supported with growth and biochemical attributes allowed us to explore key pathways that are activated if water is withheld for 15-45 days. Since earlier studies have shown that *P. bournei* seedlings are relatively more sensitive to drought stress when two years old (Chen, 2010), therefore, our experiment involved two-years old seedlings. Our results that SDW and RDW reduced significantly with the prolongation of the drought stress (**Figure 1**) imply that *P. bournei* seedlings (when two years-old) should be irrigated at least after 15d to have minimum growth reduction effects and in extreme cases, waster should be supplied within a month. Since root is the first organ to experience water deficit (Imadi et al., 2016; Yang et al., 2021), therefore the significant reduction in RDW right from 15d is understandable. The reduction in both RDW and SDW is a common drought stress response in different plant species e.g., tomato (Litvin et al., 2016) and wheat (Fang et al., 2017). The reduction in SDW also imply that drought can significantly affect timber yield and future studies should focus on this aspect from *P. bournei*’s usage point of view.

### 4.2 ROS scavenging activity increases with the increase in drought stress duration in *P. bournei*

The *P. bournei* seedlings responded to drought stress with the increasing activities of antioxidative enzymes i.e., POD, SOD, and CAT (**Figure 1**), indicating that excessive ROS were generated and reached maximum on 45d (You and Chan, 2015). These enzymes’ activities and induction of the expressions of POD, SOD, and CAT transcripts further explain that *P. bournei* seedlings initiate an antioxidant defense system from 15d. Similarly, the previous work on *P. bournei* showed that CAT, POD, and SOD activities significantly increased on 15^th^ day of drought stress (Ge et al., 2014). The treatment-specific differential expression of different POD, SOD, and CAT transcripts can be useful for the development of drought tolerant *P. bournei* genotypes. Concomitant significant increase in MDA content from 15d confirms the earlier report in *P. bournei* (Ge et al., 2014), *Pinus eliottii* (Zhang et al., 2021), and apple (Mihaljević et al., 2021). These results also support our statement that ROS levels continuously increased with the prolongation of the imposed stress. This also indicates that MDA can be used as a drought stress indicator in *P. bournei* (Zhang et al., 2021). These reports, together with our results, imply that drought can significantly affect two-years old *P. bournei* seedlings, whereas the antioxidative defense system is active throughout the studied time period i.e., 15-45d.

### 4.3 Drought stress drives expression changes in photosynthesis, chlorophyll biosynthesis, and antenna proteins in *P. bournei* leaves

**Figure 6:**
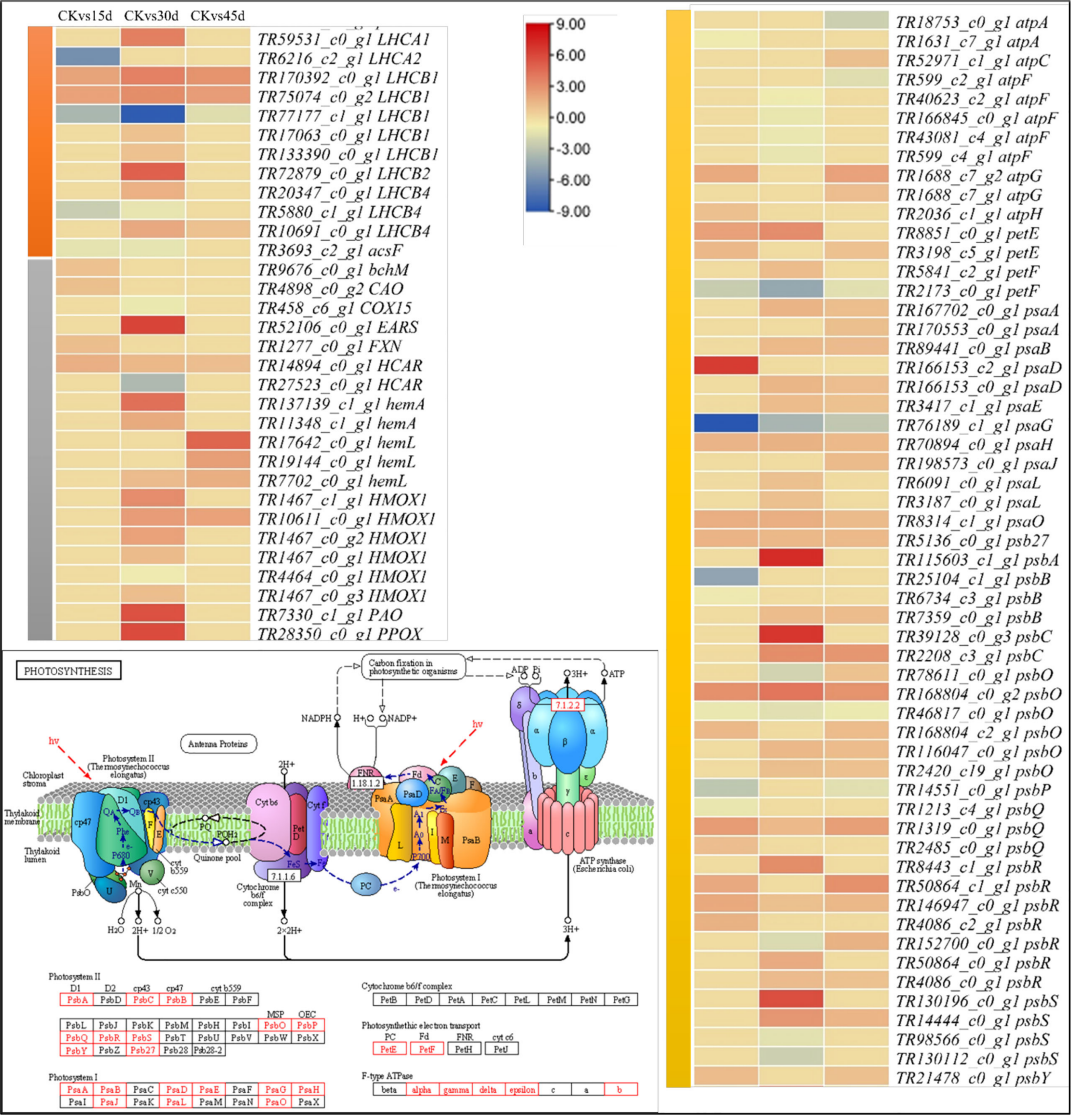
Expression changes in photosynthesis and related pathways. Heatmaps of log2FC values of the DEGs enriched in porphyrin and chlorophyll metabolism (Ko00860, grey), photosynthesis – antenna proteins (00196, orange), and photosynthesis (ko00195, yellow) pathways. The figure panel on the left bottom shows the ko00195 KEGG pathway map with the red boxes representing the enriched DEGs. The map was developed in the KEGG pathway database (Kanehisa, [[NoYear]]) using the KEGG pathway mapper (Kanehisa and Sato, 2020) by searching Ko IDs of the DEGs on respective pathways. The DEG annotation abbreviations are according to the **Supplementary Table 2**.

The differential expression of highest number of genes on 30d indicates that *P. bournei* leaves’ responses to imposed stress are on peak, which then decline on 45d as evident from lower number of DEGs in CKvs45d (**Figure 2**). The results that DEGs were significantly enriched in pathways such as photosynthesis, chlorophyll biosynthesis, antenna proteins, plant-hormone signaling, MAPK-signaling, and starch and sucrose biosynthesis indicate *P. bournei*’s responses to drought. The PSII contains the reaction centers like LHCIIs, which are affected by both the long and short-term drought stress (Vinyard et al., 2013). In Arabidopsis, it was shown that the light harvesting complexes (LHCs) disassemble from PSII, and prolongation of stressed lead to the disassembly of the PSII dimers (Chen et al., 2016). The differential expression of four PSII and one PSI transcripts indicate lower changes in PSI and PSII on 15d as compared to 30d; nine PSII related transcripts were differentially expressed (**Figure 6**; **Supplementary Table 2**). And on 45d, the expression of only four transcripts related to PSII and no expression of any PSI genes clearly shows that large-scale changes occur when stress is prolonged to 30d and by 45d the antenna proteins may have already been disassembled/destabilized as reported in rice (Dalal and Tripathy, 2018). Apart from antenna proteins, drought also significantly affects chlorophyll biosynthesis. This is evident from no expression of CAO (*TR4898_c0_g2*) in 30d and 45d, downregulation of HCAR (*TR14894_c0_g1*) on 30d and no expression on other days, and downregulation of ascF (*TR3693_c2_g1*) in 15d and 30d. These genes are present upstream the chlorophyll a/b biosynthesis (Rüdiger, 2002) (also see KEGG reference pathway map00860 on https://www.genome.jp/pathway/map00860). This in turn could affect photosynthesis as chlorophyll content is directly related with the rate of photosynthesis under drought stress (Akhkha et al., 2011). The downregulation of atpA on 15d, atpF on 30d and 45d indicates reduced ATP synthesis as compared to CK (Cheuk and Meier, 2021). This is further supported by downregulation of psaG, psbS, psbB, psbO, psbP, and psbQ transcripts in drought treated *P. bournei* leaves. Overall, based on the differential expressions, we conclude that drought stress causes changes in photosystems, reduces chlorophyll biosynthesis, and affects photosynthesis.

### 4.4 Drought stress regulates changes in metabolism and secondary metabolite biosynthesis pathways in *P. bournei* leaves

Sucrose is the main photosynthetic product in green plants, acts as energy substrate, and participates as a signal in many pathways (Du et al., 2020). Similarly, starch is biosynthesized in leaves and stored in different organs, and is mobilized under abiotic stress scenarios to provide energy and carbon and support plant growth (Krasensky and Jonak, 2012). Our results that AMY, NUDX14, EGL, scrK, glgA, SUS, otsB, and UGP2 were upregulated in response to drought stress indicate starch breakdown (Streb and Zeeman, 2012) changes in energy metabolism, cell-wall modification (Takashima et al., 2006; Nawaz et al., 2017), and sucrose level (Yang et al., 2019). Interestingly, the increased expressions of glgA and SUS are consistent with the drought stress responses in wheat (Lu et al., 2019), rice (Thomas et al., 2022), and apple (Yang et al., 2019). Thus, our results establish that *P. bournei* seedlings respond to drought stress by making changes in starch and sucrose metabolism.

Plants adapt to drought or other abiotic stress by regulating secondary metabolism. Earlier work on the understanding of the drought stress responses in plants e.g., Arabidopsis has shown that flavonoids (flavonols and anthocyanins) offer radical scavenging activities and mitigate the oxidative stress (Nakabayashi et al., 2014). Our results that multiple genes related to flavonol and anthocyanin biosynthesis i.e., F3H, CCOAMT, CHS, FLS, HCT, and C3’H were differentially regulated in drought stress (Cui et al., 2017) are important since they have been implicated as key regulators of drought stress tolerance (Cirillo et al., 2021). Of particular interest are the upregulation of C3’H in 15d and HCTs in all treatments (**Supplementary Table 2**). These genes have been previously reported to be upregulated in water deficit stress in maize (Maheswari et al., 2021) and tea leaves (Sun et al., 2018), presenting both the HCT and C3’H as good candidates for improving drought stress tolerance. Similarly, the increased expressions of bglB, F5H, REF1, and COMTs offer us a wide range of target genes for modifying drought tolerance potential in *P. bournei* (Sharma et al., 2019). The wide-spread differential regulation patterns of transcripts with same annotation observed in this study points to a need for systematic gene characterization to understand their regulation during short-term and/or prolonged drought stress. Overall, we conclude that drought stress initiates large scale transcriptomic changes in flavonoid and phenylpropanoid biosynthesis pathways in *P. bournei* leaves.

### 4.5 Drought stress drives large-scale expression changes in phytohormone signaling

Plant-hormones regulate drought-stress mediated signaling pathways. Earlier work have elucidated the positive role of auxin (IAA) in improving drought tolerance in plants (Zhang et al., 2020). The differential expression of a large number of transcripts enriched in auxin signaling pathway is indicative of significant role of this hormone in *P. bournei’s* responses to drought stress. The increased expression of multiple GH3 and SAUR transcripts in 15-45d leaves indicates auxin-signaling-driven responses in *P. bournei*. These genes are known to enhance drought stress tolerance in different plant species e.g., cotton (Kirungu et al., 2019) and Arabidopsis (Guo et al., 2018). On the other hand, the restricted growth of the seedlings after drought stress can be due to the changes in expressions of AHP, ARR-A, and ARR-B genes (Huo et al., 2020). Additionally, the most studied hormone is ABA due to its involvement in defense, growth, and development. In Arabidopsis, ~10% signaling genes are regulated by ABA (Fujita et al., 2011). The higher expression of PYLs and lower expression of PP2Cs as compared to CK indicate the possibility of ABA attachment to PYLs and degradation of PP2C. This in turn indicates probable ABA responses in leaves such as stomata closure (Bharath et al., 2021). The reduced performance of the seedlings on 15-45d of drought treatment can also be related with the downregulation of most of the BR-signaling related transcripts. This proposition is based on the fact that BR activity is linked with the abiotic stress related gene networks and complementation of BR activity can significantly restore developmental defects in BR-deficient tomato (Lee et al., 2018). Differences in the expression patterns of JA signaling related genes could hint towards cross-talk with other hormones during drought stress (Muñoz-Espinoza et al., 2015; Salvi et al., 2021). Finally, the upregulation of TGA TFs enriched in SA signaling offers useful target genes that can play role in enhancing drought tolerance in *P. bournei* (Chen et al., 2021) since TGA TFs have been implicated in drought stress tolerance (Li et al., 2019; Chen et al., 2021). Thus, overall, this study shows that drought stress induces large-scale changes in phytohormone-signaling in *P. bournei* seedlings.

### 4.6 Drought stress initiates expression changes in large number of TF families in *P. bournei* leaves

Diverse studies on multiple scenarios of drought stress tolerance together with other abiotic stresses or alone have significantly contributed towards the roles of TFs in tolerance mechanisms (Hrmova and Hussain, 2021). Since TFs have ability to bind specific DNA sequences, therefore, their roles in regulation of expression of a variety of drought responsive genes is very important (Khan et al., 2018). The result that TFs belonging to 25 different families indicates the *P. bournei* seedlings have a myriad of TFs that are drive the drought stress responses. Our results that highest number of TFs were differentially expressed on 30d are consistent with the changes in expression of large number of genes in the same treatment. The downregulation of multiple B3 DNA binding domain containing TFs, which were mostly annotated as ARFs indicates their role in growth changes during drought stress similar to tomato (Chen et al., 2021). Similarly, the downregulation of bZIP_1 TF (ABSCISIC ACID-INSENSITIVE 5-like protein 5, *TR2550_c0_g1, TR19610_c0_g1*, and *TR8244_c0_g2*) is consistent with the reduction in growth and its known function as ABA-dependent drought responses (Collin et al., 2020). In addition to these TFs, our results provide a large number of TFs from different families as candidates for studying drought stress responses in *P. bournei* and related species. Contrary to the downregulated TFs, the ones that were upregulated in the studied time periods of drought stress are also important for understanding their roles for survival of *P. bournei* seedlings under prolonged droughts.

## 5 Conclusion

This is a first report on the exploration of transcriptomic changes in *P. bournei* leaves when stressed with drought for 15-45d. We conclude that drought stress drives large scale transcriptomic changes in *P. bournei* seedlings (leaves). If drought is prolonged, the *P. bournei* seedlings exhibit continuous reduction in growth as evident from significant decrease in SDW and RDW. On molecular level, this species experiences continuous generation of ROS and damage as evident from activities of CAT, POD, and SOD, and MDA levels, respectively. As a response to drought stress, *P. bournei* initiates transcriptomic changes in signaling (hormone and MAPK pathways), metabolism (starch and sucrose biosynthesis), secondary metabolite biosynthesis, and photosynthesis related pathways. We present many candidate genes and TFs for exploring their roles in drought stress and manipulation for improving tolerance. Based on the growth attributes, enzyme activities, MDA content, and transcriptomic responses, we recommend that water withholding should not be prolonged and irrigation should be provided as early as two weeks to *P. bournei* seedlings. For future studies, we propose that rewatering experiments should be conducted to understand and confirm the relevant transcriptome signals.

## Data availability statement

The original contributions presented in the study are publicly available. This data can be found here: NCBI, PRJNA793342.

## Author contributions

Conceptualization, XL, LL, LJ, SY, YY and YuL. Data curation, SS, YaL and LJ. Formal analysis, XL, LL, SS, KD and YaL. Funding acquisition, YuL. Investigation, XL, SY and YY. Methodology, LL and SY. Project administration, LL, YaL, YY and YuL. Resources, SS and LJ. Software, YY and KD. Supervision, YuL and KD. Validation, YaL and YuL. Writing – original draft, LJ and YaL. Writing – review & editing, YY and YuL. All authors contributed to the article and approved the submitted version.

## Funding

The present study was funded by National Natural Science Foundation of China (NSFC) (41867027, 42167057, 32160223, 31860254), Outstanding Young Talent Projects of Yunnan Ten Thousand Talents Program (80201442), Key Special Program of Yunnan Province’s Science and Technology Planning Project (202201AS070028), the Science and Technology Talent Platform Program of Yunnan Provincial Science and Technology Department(202105AC160047) and General Program of Yunnan Provincial Science and Technology Department (2018FG001-039).

## Acknowledgments

We thank the Key Laboratory of National Forestry and Grassland Administration on Biodiversity Conservation in Southwest China, Southwest Forestry University, Kunming 650224, China and the Key Laboratory of Forest Disaster Warning and Control of Yunnan Province, College of Biodiversity Protection Southwest Forestry University, Kunming 650224, China.

## Conflict of interest

The authors declare that the research was conducted in the absence of any commercial or financial relationships that could be construed as a potential conflict of interest.

## Publisher’s note

All claims expressed in this article are solely those of the authors and do not necessarily represent those of their affiliated organizations, or those of the publisher, the editors and the reviewers. Any product that may be evaluated in this article, or claim that may be made by its manufacturer, is not guaranteed or endorsed by the publisher.

## Supplementary material

Supplementary Figure 1 Summary of unigene annotation of *P. bournei* leaf transcriptome sequencing.

Supplementary Figure 2 KEGG pathway enrichment analysis - scatter plots showing pathways to which DEGs were highly enriched in drought treated *P. bournei* seedling leaves as compared to control.
