# Supplementary material for: Leaf-transcriptome profiles of phoebe bournei provide insights into temporal drought stress responses
Source: Front Plant Sci. 2022 Oct 24;13:1010314. doi: 10.3389/fpls.2022.1010314 (PMC9637941; doi:10.3389/fpls.2022.1010314)
Supplement: Supplementary Figure 1 — Summary of unigene annotation of P. bournei leaf transcriptome sequencing. [file Presentation_1.pptx]

## Slide 1
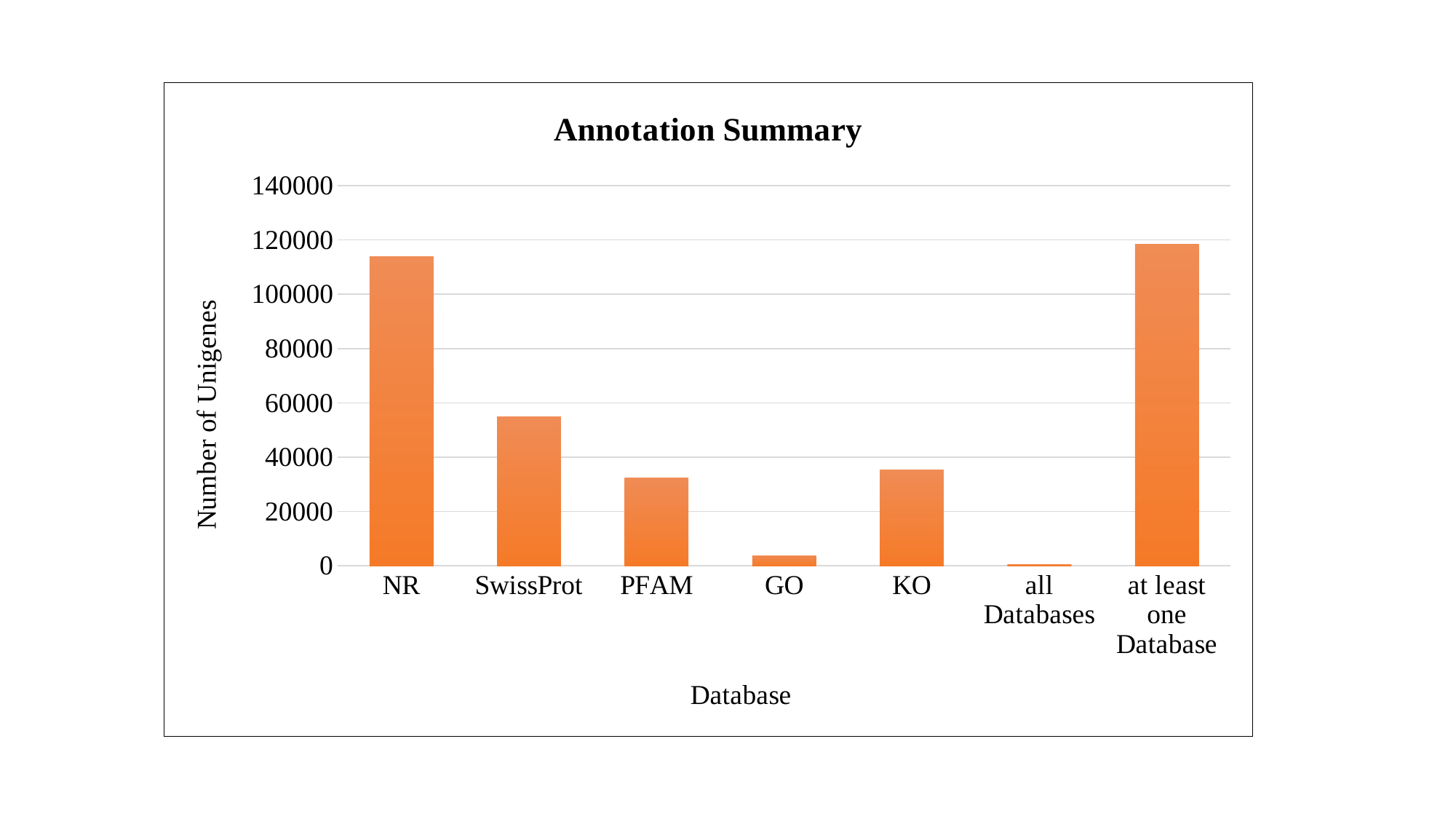

### Chart: Annotation Summary
| Category | Number of Unigenes |
|---|---|
| NR | 113841.0 |
| SwissProt | 54750.0 |
| PFAM | 32413.0 |
| GO | 3797.0 |
| KO | 35226.0 |
| all Databases | 560.0 |
| at least one Database | 118292.0 |
