# Supplementary figures and images for: Leaf-transcriptome profiles of phoebe bournei provide insights into temporal drought stress responses
Source: Front Plant Sci. 2022 Oct 24;13:1010314. doi: 10.3389/fpls.2022.1010314 (PMC9637941; doi:10.3389/fpls.2022.1010314)

## Slide 1
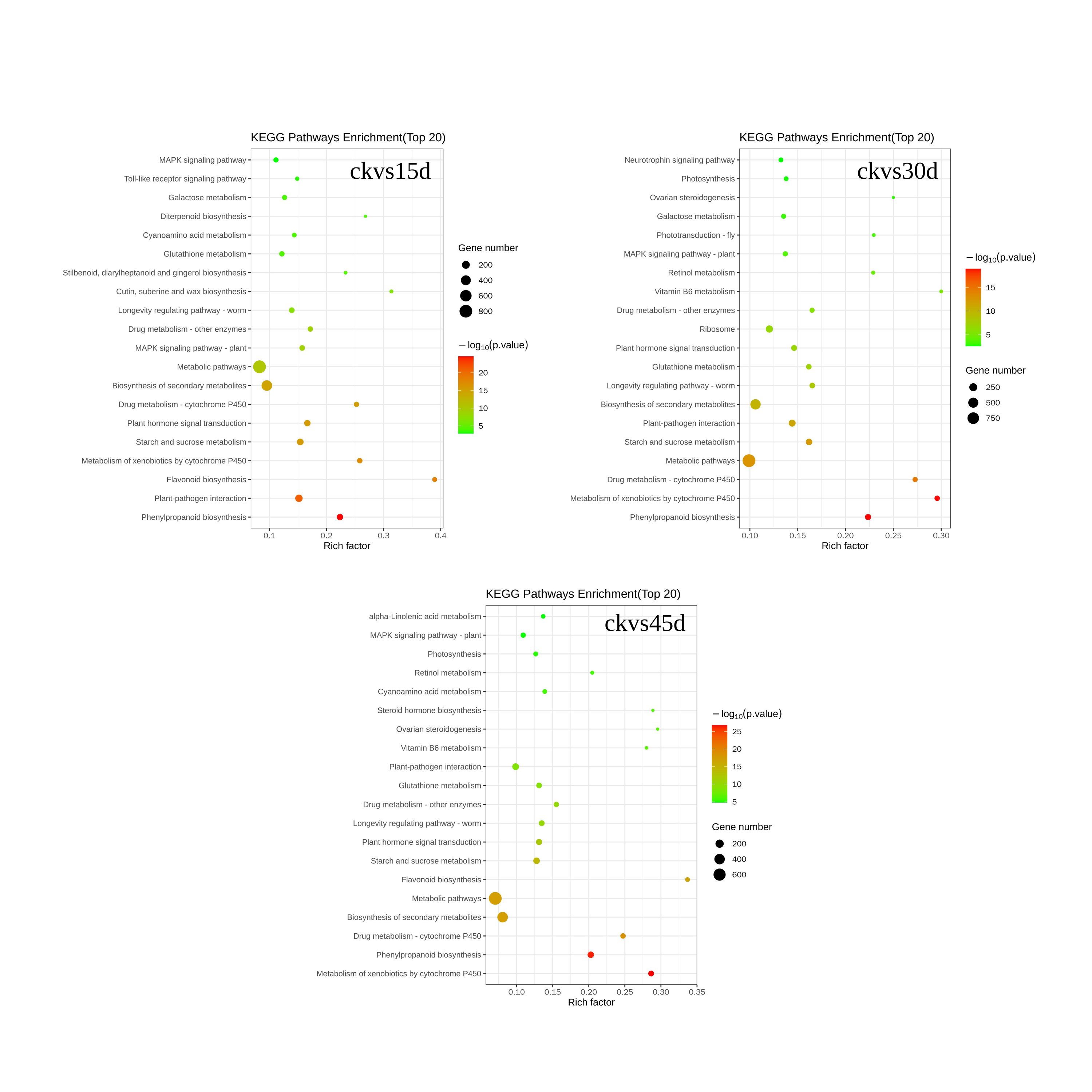

ckvs15d
ckvs30d
ckvs45d

Supplement: Supplementary Figure 2 — KEGG pathway enrichment analysis - scatter plots showing pathways to which DEGs were highly enriched in drought treated P. bournei seedling leaves as compared to control. [file Presentation_2.pptx]
